# Development of a normal tissue complication probability (NTCP) model for radiation-induced hypothyroidism in nasopharyngeal carcinoma patients

**DOI:** 10.1186/s12885-018-4348-z

**Published:** 2018-05-18

**Authors:** Ren Luo, Vincent W. C. Wu, Binghui He, Xiaoying Gao, Zhenxi Xu, Dandan Wang, Zhining Yang, Mei Li, Zhixiong Lin

**Affiliations:** 1 Department of Radiation Oncology, Cancer Hospital of Shantou University Medical College, 7 Raoping Road, Shantou, 515000 Guangdong China; 2 Department of Radiation Oncology, Medical Center - University of Freiburg, Freiburg, Germany; 3 Department of Health Technology and Informatics, Hong Kong Polytechnic University, Hong Kong, China

**Keywords:** Radiation-induced hypothyroidism, Normal tissue complication probability, Nasopharyngeal carcinoma, Radiotherapy, Late complication

## Abstract

**Background:**

The objectives of this study were to build a normal tissue complication probability (NTCP) model of radiation-induced hypothyroidism (RHT) for nasopharyngeal carcinoma (NPC) patients and to compare it with other four published NTCP models to evaluate its efficacy.

**Methods:**

Medical notes of 174 NPC patients after radiotherapy were reviewed. Biochemical hypothyroidism was defined as an elevated level of serum thyroid-stimulating hormone (TSH) value with a normal or decreased level of serum free thyroxine (fT4) after radiotherapy. Logistic regression with leave-one-out cross-validation was performed to establish the NTCP model. Model performance was evaluated and compared by the area under the receiver operating characteristic curve (AUC) in our NPC cohort.

**Results:**

With a median follow-up of 24 months, 39 (22.4%) patients developed biochemical hypothyroidism. Gender, chemotherapy, the percentage thyroid volume receiving more than 50 Gy (V_50_), and the maximum dose of the pituitary (P_max_) were identified as the most predictive factors for RHT. A NTCP model based on these four parameters were developed. The model comparison was made in our NPC cohort and our NTCP model performed better in RHT prediction than the other four models.

**Conclusions:**

This study developed a four-variable NTCP model for biochemical hypothyroidism in NPC patients post-radiotherapy. Our NTCP model for RHT presents a high prediction capability.

**Trial registration:**

This is a retrospective study without registration.

**Electronic supplementary material:**

The online version of this article (10.1186/s12885-018-4348-z) contains supplementary material, which is available to authorized users.

## Background

Radiation-induced hypothyroidism (RHT) is one of the common late complications in patients receiving radiotherapy to the neck region, which could be subclinical or clinical. The subclinical hypothyroidism (HT), in the presence of an elevated serum thyroid-stimulating hormone (TSH) concentration and normal serum free thyroxine (fT4) and/or serum free triiodothyronine (fT3) concentration, can only be diagnosed by a laboratory test. The clinical HT which is characterized by an elevated TSH concentration and decreased fT4 and/or fT3 concentration is more overt with fatigue, cold intolerance, weight gain, constipation and dry skin as the major clinical manifestations. The incidence of radiation-induced clinical HT is 6–20% of head and neck cancer patients, while the incidence of subclinical HT is higher, ranging from 24 to 50% [1].

Nasopharyngeal carcinoma (NPC) is an epidemic cancer in Southern China. Irradiation to bilateral necks is a regular practice since the disease presents with a high incidence of neck lymphadenopathy of over 75% [2], resulted in unavoidable irradiation with relatively high dose to part of the thyroid gland. Previous studies have reported the relatively high incidence of RHT (22.1% to 33.7%) in post-radiotherapy NPC patients [3–6]. Since the life span expectation of NPC patients has remarkably improved in the last decade due to the emerging of effective chemotherapy and advanced radiotherapy techniques [7], the demand for reducing the radiation-induced side effects including RHT has been increasing.

Clinical characteristics included gender [3, 8, 9], age [3, 10–12], neck surgery [13] and early T stage [3, 11] were considered as risk factors of RHT. Nowadays, dose-volume data is available by retrieving from the commercial treatment planning system and the parameters such as the thyroid minimum dose (D_min_), the thyroid mean dose (D_mean_), and the percentage of thyroid volume receiving more than 30, 40, 45, 50 Gy [4, 10–12, 14–20] were identified to be dosimetric predictors for RHT. Therefore, establishing a normal tissue complication probability (NTCP) model based on both clinical and dose-volume factors may offer an effective method of predicting RHT [21]. Two NTCP models [16] for RHT based on Hodgkin’s lymphoma patients have been reported. Another two NTCP models based on the thyroid mean dose and thyroid volume have also been built for head and neck cancer patients excluding NPC [14, 17].

Unlike non-NPC cases, the dose to thyroid and pituitary glands are relatively high in most NPC patients. Therefore, it remains doubtful whether the NTCP models [14, 16, 17] for RHT based on non-NPC patients are applicable for NPC patients. We conducted this study to establish a NTCP model for RHT in NPC survivors and evaluated its performance by comparing it with four existing models.

## Methods

The detail information of patient eligibility, radiation therapy, chemotherapy regimen, and thyroid function evaluation was also described in our previous publications [4, 22].

### Patient eligibility

Patients’ records from an ongoing prospective study [4] of post-radiotherapy thyroid changes in NPC patients were reviewed. Patients with normal baseline thyroid hormone levels (normal TSH, fT3, and fT4) before radiotherapy, without previous and present thyroid dysfunction, and without receiving electron boost or neck surgery to residual lymph nodes were eligible. One hundred and seventy-four newly diagnosed NPC patients in Cancer Hospital of Shantou University Medical College, which were recruited from December 2007 to February 2015, met the criteria and were included in this retrospective study. They were all re-staged using the 7th edition of American Joint Committee on Cancer (AJCC) staging system. This study was approved by our center’s institutional review board.

### Radiation therapy and dosimetric analysis

For all patients, CT scans (CT scanner: Philips Brilliance CT Big Bore Oncology Configuration, Cleveland, OH) were performed in the supine position with intravenous contrast using a 3 mm slice thickness from the head to 2 cm below the sternoclavicular joint. The CT images were then transferred to Pinnacle 7.6c treatment planning system (TPS) (Pinnacle^3^, Philips, Eindhoven, The Netherlands), on which the target volumes and the organs at risk (OARs) including thyroid and pituitary glands were contoured manually.

In NPC radiotherapy planning, the gross tumor volume (GTV) included the primary disease (GTVnx) and the lymph nodes (GTVnd). The clinical target volume of the nasopharynx (CTVnx) included the anatomic areas at risk of microscopic invasion [23]. The CTV of the lymph nodes (CTVnd) included lymph nodes at II to V levels. High-risk CTV (CTV1) indicated soft tissue adjacent to GTV and involved lymphatic drainage regions. Low-risk CTV (CTV2) included the bilateral II, III, and VA for N0 cases and ipsilateral IV, VB for the cases with levels II and/or III involved The planning target volume (PTV) was established by adding 3–5 mm to the CTV.

Dose prescription to PTVs was stated as follows: 70Gy to the PTVnx in 2.0 Gy per fraction for 3-dimensional conformal radiotherapy (3D-CRT), or 2.12 to 2.33 Gy per fraction for intensity-modulated radiotherapy (IMRT); 66 Gy to the PTVnd; 60 Gy to the PTV1 of high-risk CTV1 and 54 Gy to the PTV2 of low-risk CTV2. The dose constraints of normal tissue: the maximum dose (D_max_) of the brainstem, optic nerves, optic chiasm, spinal cord, lens was 54 Gy, 54 Gy, 54 Gy, 45 Gy, and 5 Gy, respectively; D_mean_ of parotid gland, oral cavity and larynx was 35 Gy, 38Gy and 38 Gy, respectively. No specific dose constraints were performed to constrictors, pituitary and thyroid glands.

No patients underwent re-CT and re-planning during radiotherapy. The dosimetric data was generated from TPS, including D_min_, D_max_, and D_mean_ to thyroid; the maximum and mean dose to pituitary (P_max_ and P_mean_); the percentage of thyroid volume receiving more than X Gy (V_x_) in step of 5 Gy from 10 to 60 Gy; the absolute volume receiving more than 30 Gy (V_30_ cc) of thyroid and the absolute thyroid volume.

### Chemotherapy

The neoadjuvant chemotherapy regimen was one cycle of docetaxel 75 mg m^− 2^ on Day 1 and two cycles of cisplatin 75 mg m^− 2^ on Days 1 and 22. Concurrent chemotherapy consisted of cisplatin 75 mg m^− 2^ on Day 1 and 96 h of continuous infusion fluorouracil 750 mg m^− 2^/day every 3 weeks for two cycles. Adjuvant chemotherapy regimen was the same as the regiment of concurrent chemotherapy, but it was administered every 3 weeks for two cycles post-radiotherapy.

### Thyroid function evaluation

Thyroid function included TSH, fT3, and fT4 levels were evaluated pre-radiotherapy and at each follow-up visit. The electrochemiluminescence method by Elecsys 2010 analyzer (Hitachi High Technology Corporation, Tokyo, Japan) was used, with the reference ranges of 0.27–4.20 μIU/mL, 3.1–6.8 pmol/L, and 12.0–22.0 pmol/L, respectively. The reproducibility of the three thyroid hormones tests was acceptable, and their coefficient variations ranged between 1.3% and 1.5%. After radiotherapy, thyroid hormones tests were performed every 3 months during the first year, every 6 months in the second to the fifth year and annually after that. Biochemical HT is TSH value > 4.20 μIU/mL with the low or normal level of fT4. Patients whose fT3 and fT4 were at low levels and with low or normal TSH level, would be diagnosed as central HT.

### Statistical analysis

The endpoint of this study was biochemical HT. In NTCP pre-modeling process, Spearman’s rank correlation coefficients were calculated to access the correlation between different variables, and the correlation between different variables and the biochemical HT using SPSS 21.0 software (SPSS, Inc., Chicago, IL, USA). If the Spearman’s rank correlation coefficient was greater than 0.85 between two variables, the one with the lower correlation with biochemical HT would be excluded from NTCP modeling to avoid model overfitting [16]. Univariate logistic regression analyses for both clinical and dose-volume factors were also applied using SPSS 21.0 software. The NTCP modeling was performed by an open-source tool (Dose Response Explorer System [21]) and based on the multivariable logistic regression formula:

$$ \mathrm{NTCP}={\left(1+{\mathrm{e}}^{\hbox{-} \mathrm{S}}\right)}^{\hbox{-} 1} $$

where S = β_0_ + β_1_x_1_ + β_1_x_1_ + … + β_m_x_m._ x_1_, x_2_. .. x_m_ are different input parameters, and β_0_, β_1_ ... β_m_ are the logistic regression coefficients of corresponding input parameters.

In modeling process, the variable chemotherapy was coded 0 (not receiving chemotherapy) and 1 (receiving chemotherapy); the variable gender was coded 1(female) and 0 (male). First, the optimal model order was estimated by leave-one-out cross-validation (2000 samples). Second, the model parameters were estimated by multivariate logistic regression analysis with the forward selection with 1000 bootstraps. The goodness-of-fit of our NTCP model was evaluated by the Hosmer-Lemeshow test and visualized in an octile plot. In octile plot, patients were uniformly binned into 8 groups based on the predicted risk of biochemical HT by our model.

The regression coefficients of four existing logistic regression based NTCP models: Boomsma et al., Cella model 1 et al., Cella model 2 et al., and Rønjom et al. [14, 16, 17] were given in Table 1. Model performance was evaluated by the area under the receiver operating characteristic curves (AUC). Firstly, a comparison of AUCs of each model in its own cohort was performed using z-test. Secondly, the AUCs of these five models in our NPC cohort were calculated and compared using the method suggested by Delong et al. [24] using MedCalc 11.4.2.0 (MedCalc, Mariakerke, Belgium). The comparing method for ROC by DeLong et al. is a nonparametric method that does not require the assumption of normality. For this reason, this method has become the most widely used one in practice. A *p*-value of < 0.05 was considered statistically significant.

**Table 1:** Five normal tissue complication probability (NTCP) models from four published and present study

NTCP model	b	constant	OR	95% CI	AUC
Boomsma et al.		0.011			0.85 (95% CI: 0.78–0.92)
D_mean_	0.062		1.064/Gy	1.029–1.101	
Thyroid volume	−0.19		0.826/cm^3^	0.740–0.921	
Cella et al. model 1		−1.83			0.865 (95% CI: 0.793–0.945)
V30%	0.038		1.039	1.019–1.059	
Gender	−2.32		0.098	0.019–0.500	
Cella et al. model 2		1.94			0.874 (95% CI: 0.750–0.951)
V30 cc	0.26		1.297	1.087–1.547	
Gender	−2.21		0.110	0.021–0.580	
Thyroid volume	−0.27		0.763	0.615–0.947	
Rønjom et al.		−2.019			NA
D_mean_	0.0821		1.12/Gy	1.07–1.20	
Thyroid volume	−0.189		0.75/cm^3^	0.64–0.85	
Luo et al.		−2.695			0.793(95% CI: 0.725–0.851)
V_50_	0.050		1.051	1.027–1.076	
P_max_	−0.026		0.974	0.956–0.993	
Gender	1.280		3.598	1.461–8.865	
Chemotherapy	2.902		18.213	1.920–172.766	

Abbreviation: *OR* odds ratio, *CI* confidence interval, *AUC* the area under the receiver operating characteristic curve, *NA* not available in original article

## Results

One hundred and seventy-four patients (129 males and 45 females) with a median age of 49.5 (range, 16 to 69) years old were included in this study. Thirty-two (18.4%) patients were treated with 3D-CRT while the other 142 (81.6%) with IMRT. The incidences of RHT in patients who received neoadjuvant plus concurrent chemotherapy, only concurrent chemotherapy, and concurrent chemotherapy plus adjuvant chemotherapy were 19.44% (7 of 36 patients), 25.64% (30 of 117 patients), and 50% (1 of 2 patients). One of 19 patients who did not received chemotherapy experienced RHT (5.26%). Additional information about patient characteristics and treatment is shown in Table 2.

**Table 2:** Patient characteristics and the correlation between clinical factors and RHT

		Univariate	Multivariate		
Variables	n(%)	*p* value	*p* value	OR	95% CI
Numbers	174				
Gender		0.005	0.005	3.598	1.461–8.865
Female	45 (25.9)				
Male	129 (74.1)				
Age (years)		0.066			
Median	49.5				
Range	16–69				
T stage		0.170			
1–2	64 (36.8)				
3–4	110 (63.2)				
N stage		0.329			
0	16 (9.2)				
1–3	158 (90.8)				
Stage		0.174			
I-II	20 (11.5)				
III-IV	154 (88.5)				
Technique		0.393			
3D-CRT	32 (18.4)				
IMRT	142 (81.6)				
Chemotherapy		0.091	0.011	18.213	1.920–172.766
No	19 (10.9)				
Yes	155 (89.1)				

Abbreviation: *OR* odds ratio, *CI* confidence interval

**Fig. 1:**
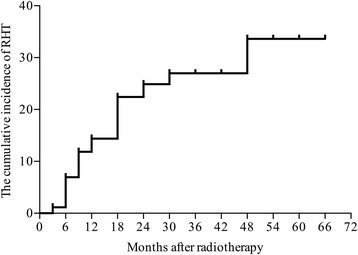
The Kaplan-Meier curve for the cumulative incidence of biochemicalhypothyroidism

After a median follow-up of 24 (range, 3 to 66) months, biochemical HT occurred in 39 (22.4%) patients. The mean values of P_mean_ and P_max_ of pituitary gland were 30.366 (range, 2.758 to 72.274) Gy and 37.856 (range, 3.735 to 74.070) Gy, respectively, but no patients were diagnosed as central HT. The estimated incidences of RHT were 14.4%, 22.4% and 24.9% at 12, 18 and 24 months, respectively (Fig. 1). The median latency of biochemical HT was 9 (range, 3 to 48) months after radiotherapy and 37 (94.9% of 39) patients developed biochemical HT within 24 months.

**Fig. 2:**
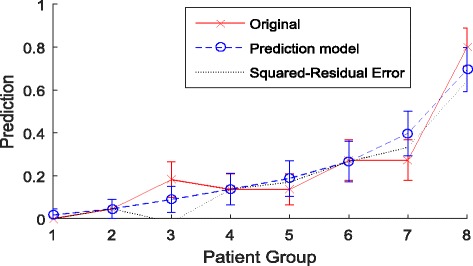
The octile plot displays the goodness-of-fit of our NTCP model

The cross-correlation matrixes of parameters were showed in Additional file 1: Table S1. High correlations (correlation coefficient > 0.85) were found between dose-volume factors but not in clinical factors. As a result, most of the dosimetric factors except D_max_, V_50_, V_60_, and P_max_ were excluded from NTCP modeling process to avoid overfitting. Thus, only clinical factors and D_max_, V_50_, V_60_, and P_max_ were taken into the modeling process. At first, the model order was determined to be four by leave-one-out cross-validation method. Subsequently, in model parameters estimation, gender, chemotherapy, V_50_, and P_max_ of thyroid gland were identified as the optimal parameters of this four-variable NTCP model for biomedical HT (Table 1). The results of univariate and multivariate analysis of dosimetric variables are showed in Table 3. The Hosmer-Lemeshow test (*p* = 0.306) and the octile plot (Fig. 2) show goodness-of-fit of our NTCP model.

**Table 3:** Univariate and multivariate analysis of dosimetric variables

			Univariate	Multivariate		
Variables	mean	SD	*p* value	*p* value	OR	95% CI
Volume (cc)	17.324	8.403	0.703			
D_min_ (Gy)	19.579	12.768	0.016			
D_mean_ (Gy)	42.417	10.145	0.001			
D_max_ (Gy)	65.500	5.819	0.536			
V_x_(%)						
V_10_	91.111	16.341	0.124			
V_15_	88.863	19.032	0.128			
V_20_	86.660	21.072	0.093			
V_25_	84.135	23.041	0.070			
V_30_	80.583	25.409	0.038			
V_35_	74.270	25.425	0.011			
V_40_	62.308	23.050	0.001			
V_45_	50.496	21.220	0.000			
V_50_	38.915	19.477	0.000	0.000	1.051	1.027–1.076
V_55_	24.598	16.243	0.000			
V_60_	12.111	12.856	0.006			
V_30_cc	14.044	8.133	0.730			
P_mean_ (Gy)	30.366	22.712	0.072			
P_max_ (Gy)	37.856	23.719	0.054	0.007	0.974	0.956–0.993

Abbreviation: *SD* standard deviation, *OR* odds ratio, *CI* confidence interval

**Fig. 3:**
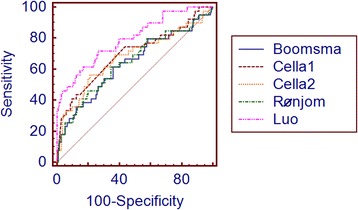
Receiver operating characteristic curves (ROC) of five biochemical hypothyroidism NTCP models in NPC cohort

Because the AUC was unavailable in Rønjom et al.’s article [17], the AUCs of the rest four models in their corresponding patient cohort were compared. There was no significantly statistical difference between our NTCP model and the other three models (Table 4). AUCs of five models were calculated based on our NPC cohort. Compared to the other four AUCs, the AUC of Luo et al. model was statistically higher [14, 16, 17] (Table 4). The receiver operating characteristic curves of the five models are depicted in Fig. 3.

**Table 4:** Comparision of five models using AUC

	AUC*	95% CI	p^‡^	AUC^†^	95% CI	p^§^	p^¶^
Boomsma	0.85	0.78–0.92	0.134	0.636	0.560–0.708	0.022	0.008
Cella model 1	0.865	0.793–0.945	0.082	0.694	0.620–0.762	0.001	0.030
Cella model 2	0.874	0.750–0.951	0.159	0.677	0.602–0.746	0.003	0.024
Rønjom	NA	NA	NA	0.646	0.570–0.717	0.014	0.011
Luo	0.793	0.725–0.851		0.793	0.725–0.851	0.000	

AUC^*^, area under the receiver operating characteristic curve calculated in each own cohort

AUC^†^, area under the receiver operating characteristic curve calculated in NPC cohort

p^‡^, p-value evaluated in each own cohort respect to *Luo* et al. *model*

p^§^, p-value of each AUC^†^

p^¶^, p-value evaluated in NPC cohort respect to *Luo* et al. *model*

NA, not available

## Discussion

Hypothyroidism is a common side effect caused by radiotherapy in NPC patients. However, in China, thyroid function tests are not routine clinical practice neither before nor after radiotherapy for NPC patients. In this study, the incidence of biochemical HT after radiotherapy is 23.9%, which is similar to other NPC studies [3, 4, 6, 12]. We found the latency of biochemical HT is short (median 9 months) and most of the biochemical HT (89.7%, 35 in 39 patients) developed within 18 months post-radiotherapy. The previous studies also showed that the peak incidence of RHT was 1 to 2 years after radiotherapy [10, 14, 15, 25–27]. The incidence of RHT will further increase within a longer follow-up period [28] and the patients with subclinical HT will develop clinical HT in the future follow-up [6]. Therefore, we suggest regular follow-up for thyroid function in the first two years post-radiotherapy is particularly important and the further follow-up (even lifelong) thereafter is also necessary for the patients who received cervical radiation.

In accordance with the previous studies [3, 8, 9, 16, 19, 22], the female gender and V_50_ were identified as risk factors in this study. It remains controversial [9, 29–31] whether chemotherapy is a predictor of RHT. However, in our previous study [22], chemotherapy plays a major role in the nomogram for biochemical HT prediction in NPC patients. In this study, only one experienced biochemical HT among 19 patients who did not receive chemotherapy, and chemotherapy was identified as an independent predictor in the multivariate analysis. The P_max_ was found as a protective factor of biochemical HT, which might due to the radiation effect to the pituitary gland by preventing the TSH increasing [3].

Cella et al. [16] reported two NTCP models in Hodgkin’s lymphoma patients respectively based on thyroid V_30_ plus gender, and V_30_cc, gender plus thyroid volume (Table 1). The initial thyroid volume and D_mean_ based NTCP models were built in two studies [14, 17] of non-NPC head and neck cancer patients (Table 1). In this study, we develop a NTCP model based on gender, chemotherapy, V_50_, and P_max_. All these five studies used the same endpoint (biochemical HT) and uncorrected dosimetric parameters to construct the NTCP models, which made them comparable.

Firstly, when the model performance of the four models was evaluated in their respective cohorts, the difference between our NTCP model and the other three models was not significant. Secondly, the model performance of the five models was calculated and compared in our NPC cohort, and we found the performance of Luo et al. model was significantly better than the other four models [14, 16, 17] that were derived from non-NPC patients. Models derived from different patient populations and the NPC patients being the external cohort for the other four models may result in decreasing performance in non-NPC models. However, a main difference from other models was that Luo et al. model included the dosimetric factor of pituitary which might help to improve the model performance in predicting biochemical HT in NPC patients. Therefore, considering the effect of radiation on both the pituitary and thyroid glands might be necessary for modeling NTCP model for RHT in NPC patients. The thyroid volume was not identified as a predictive factor in this study, which might be the possible reason for the relatively low performance of two NTCP models which were based on thyroid volume [14, 17] in this cohort.

Lo Galbo et al. [32] and Lin et al. [33] revealed that autoimmune response was correlated to RHT. To improve the performance of NTCP models for RHT, it is considerable to introduce thyroid autoimmune factors such as thyroperoxidase antibody and thyroglobulin antibody into the modeling process. In this regard, it may be rational to add these two immunologic tests to the regular follow-up for patients who received irradiation to the neck areas.

There were still some limitations to this study. The median follow-up (24 months) was relatively short, which may make a negative impact on estimating long-term risk on RHT with our NTCP model. In a recent study [6], pituitary dosimetric factors have been found not correlated with either biochemical or clinical HT. Thus, a prospective-designed study with longer follow-up period is necessary to validate the importance of the effect of irradiation on the pituitary gland and our NTCP model.

## Conclusions

We have developed a four-variable NTCP model for RHT in NPC patients post-radiotherapy and demonstrated its high prediction capability.

## Additional file

Additional file 1: Table S1. Spearman’s rank self-correlation matrix of clinical and dosimetric variables. (XLS 26 kb)

## Abbreviations

3D-CRT: 3-dimensional conformal radiotherapy
AJCC: American Joint Committee on Cancer
AUC: Area under the receiver operating characteristic curve
CTVnd: The lymph nodes of the clinical target volume
CTVnx: The clinical target volume of the nasopharynx
D_max_: The maximum dose
D_mean_: The thyroid mean dose
D_min_: The thyroid minimum dose
fT3: Serum free triiodothyronine
fT4: Serum free thyroxine
GTV: The gross tumor volume
GTVnd: The lymph nodes of the gross tumor volume
GTVnx: The primary disease of the gross tumor volume
HT: Hypothyroidism
IMRT: Intensity-modulated radiotherapy
NPC: Nasopharyngeal carcinoma
NTCP: Normal tissue complication probability
OARs: Organs at risk
P_max_: The maximum dose of the pituitary
PTV: The planning target volume
RHT: Radiation-induced hypothyroidism
TPS: Treatment planning system
TSH: Serum thyroid-stimulating hormone
V_30_cc: The absolute thyroid volume receiving more than 30 Gy
V_50_: The percentage thyroid volume receiving more than 50 Gy
V_x_: The percentage of thyroid volume receiving more than X Gy
